# Conservative treatment of Fibrous Dysplasia

**DOI:** 10.12669/pjms.35.3.14

**Published:** 2019

**Authors:** Besime Ahu Kaynak

**Affiliations:** 1 *Besime Ahu Kaynak, Assistant Professor, School of Health Sciences, Toros University, Mersin, Turkey*

**Keywords:** Fibrous dysplasia, Mandible, Conservative treatment

## Abstract

Fibrous Dysplasia (FD) is a developmental disorder of bone that can affect one bone (monostotic type) or multiple bones (polyostotic type). The disease can be associated with hyperpigmentation and endocrinological disorders. In general, FD is found in teenagers, in the second decade of life. It often involves the long bones, craniofacial bones, ribs and pelvis. Approximately 30% of monostotic FD (MFD) lesions are found in the cranial, rather facial bones. It frequently appears in the posterior region of the jaw bone and is usually unilateral. The etiology of FD is not clear but even so genetic predisposition is suspected. The diagnosis is based on radiological and histopathological examination.

We present an unusual case of symptomatic FD. A 15-year-old female patient was admitted to the clinic, complaining gradually increased swelling on her left side of the mandible one year ego with severe and unusual pain at related region. The bony enlargement was extending from median mandible to the crestal marginal level. Surgical shaving and recountering of mandible through the delicate preservation of the mental nerve in the left side of mandible was carried out.

The aim of this report is to present the conservative treatment as sufficient treatment modality for the treatment of FD during puberty.

## INTRODUCTION

Fibrous dysplasia is a slowly progressive, benign, rare and idiopathic skeletal disorder in which normal bone and marrow are replaced by fibrous tissue and randomly distributed woven bone, usually with pain, bony deformity and pathologic fractures.^1^ In 1938 had Lichtenstein first coined the term ‘’fibrous dysplasia (FD)’’^2^ Mutation in the guanine nucleotide-binding protein coding gene in early stages of life is responsible in the etiology of the disease. A cell population with this genetic feature, which is not capable of producing normal tissue, instead generates a substitute of disorganized woven bone.^3^

FD involves often the long bones, craniofacial bones, ribs and pelvis. It accounts for about 2.5% of all bone tumors and 7.5% of the benign bone neoplasms. The lesion has mainly two forms; monostotic form defines single bone involvement and polyostotic form defines multiple bone involvement. Approximately 30% of monostotic FD (MFD) lesions are found in teenagers, and it usually becames static after adulthood.^4^ FD insolves the maxilla almost two times more often than mandible. It frequently appears in the posterior region of the jaw bone and is usually unilateral. The craniofacial variety of FD is a localized form of this pathology characterized by confluent involvement of adjacent bones of the cranium and skull base. It is a disease that functionally and aesthetically cripples the affected person.^5^

Cases of FD showing rapid growth with extensive bone destruction during childhood are rare. Aspecial form of FD is MCCune-Albright syndrome, which is characterized by endocrine dysfunction including acromegaly, Cushing syndrome, hyperthyroidism and vitamin D resistant rickets. The most common features of this syndrom are; precocious puberty in girls and brownish pigmentations of the skin (cafe-au-lait spots) with irregular borders.^6^ Depending on it’s location, the signs and symptoms can vary from an asymptomatic facial deformity to more grave consequence like vision changes, hearing impairment and nasal obstruction.^7^

The aim of this article was to present an interesting case of conservative treatment through submandibular approach for the treatment of fibrous dysplasia during puberty.

## CASE REPORT

A 15-year-old female patient was admitted to the department of oral & maxillofacial surgery, complaining of gradually increasing swelling on her left side of mandible, started one year ago with severe and unusual pain at the related region. The patient declined to history of any previous toothache and trauma to the affected site. The review of systems was non-contributory. The past medical and dental histories were unremarkable. Upon examination the patient was moderately built and had a normal intellect.

**Fig.1:**
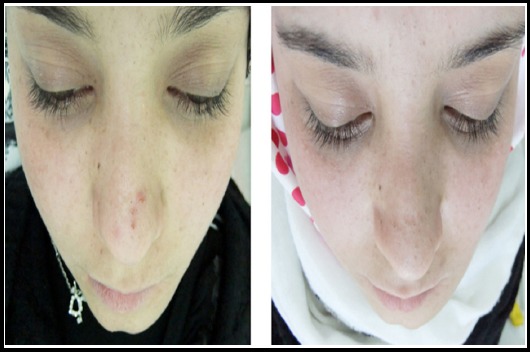
Extraoral photograph showing diffuse swelling and facial asymmetry over the left side of mandible.

The intraoral examinaton revealed a bony enlargement extending from median mandible to the retromolar region, and inferior border of the mandible to the crestal marginal level. The depth of the left vestibul sulcus was thoroughyl decreased due to hard bony expansion. The extraoral examination revealed hard uniform and large expansile mass in the left side of the mandible. Facial asymmetry was present. Left submandibular lymph nodes were impalpable and insensitive in palpation (Fig.1).

**Fig.2:**
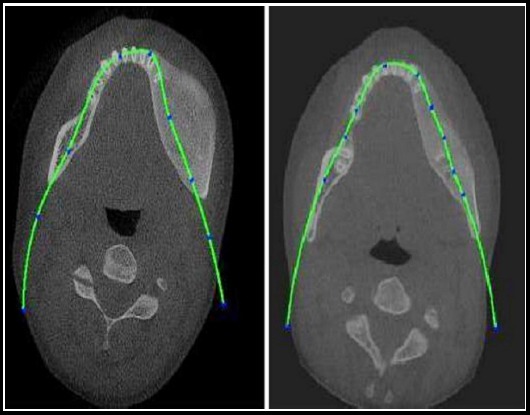
Dental CT showed diffuse increase through out of the lesion of mandible.

Conventional radiographs and computerized tomographic scan showed diffuse increase of the lesion of mandible, with loss of normal trabecular pattern leading to classical ground glass pattern (Fig.2). The CT scans were performed on a multislice spiral CT unit (Somatom Definition Edge; Siemens, Erlangen, Germany). The exposure parameters were tube voltage -120 kV, tube current -270 mA, and slice thickness -1mm. The axial section CT image of mandible showed expansion of the body of the mandible with few lytic areas bilaterally but greater in degree on the left side and expansion of the left ramus. Ground glass appearance of the bone was clearly appreciable. Laboratory investigations revealed slight rise in erythrocyte sedimentation rate (ESR) and mild change in the alkaline phosphotase level which was approximately 566 units.

The position of the mandibular canal was analyzed through the axial CT, which showed that the vertical distance between crestal margin and inferior alveolar nerve at mental foraminal level on the right and left side of the lesion was 19.06 and 23.50 mm respectively. The horizontal distance between alveolar inferior nerve and other cortex of the mandible at lower first molar region on the right and left side was 5.25 and 15.50 mm respectively.

**Fig.3:**
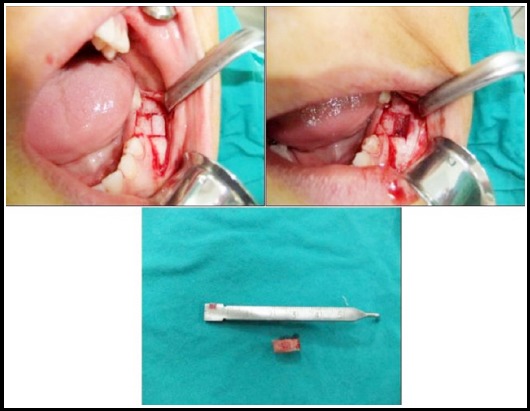
Incisional biopsy for histopathological examination.

Two separate interventions through incisional biopsy were made for histopathological examinations. The first histopathological specimen revealed increase in mitotic activity, presence of osteoblastic chain surrounding bone trabecules, which lead us to suspect the lesion as being ossifying fibroma. It also lead us to perform second incisional biopsy. A clinical and radiographic diagnosis of FD was confirmed with pathologic examinatons of the specimen examination (Fig.3).

The crestal and sulcular incision was made extending from posterior edentulous area to the median mandible with vertical releasing incision in anterior mandible. Surgical shaving and recountering of mandible through delicate preservation of the mental nerve in the left side of the mandible was performed. Mucoperiosteal elevation was made with identification of the margin of mental nerve. The expanded bone was removed throughout the lesion excluding the bone surrounding the mental foramina. Then gradual osteotomy was performed by chiesel and mallets according to the anatomy of mantel foramina which was analysed via computerized tomography. Horizontally, 8-9mm of bone surgical shaving was performed symetrically with the reference of right mandibular region. CT displayed a vital role for identification of the mental foramina during osteotomy especially in the bone surrounding mental nerve bundle. The surgical technique included intersecting with oscilating saw, performing osteotomy by chiesels, recontouring with big round burs and rasping with a bone file. Mucosal healing was uneventful. Facial esthetic lines were obtained in the evaluation from the frontal aspect. In evaluation from the lateral profile, bone expansion at inferior border of the left mandible was not eliminated due to staying away from invasive technique (extraoral approach) during pubertal phase.

**Fig.4:**
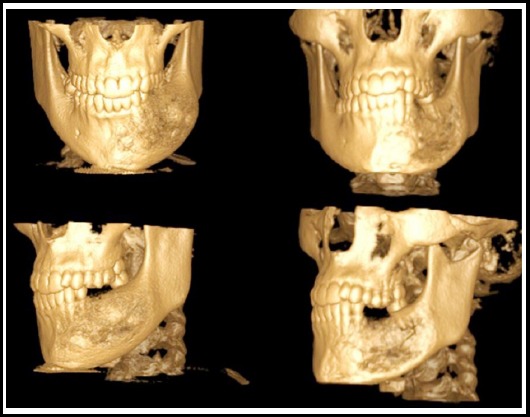
3D Reconstruction of the mandible showing perforation.

**Fig.5:**
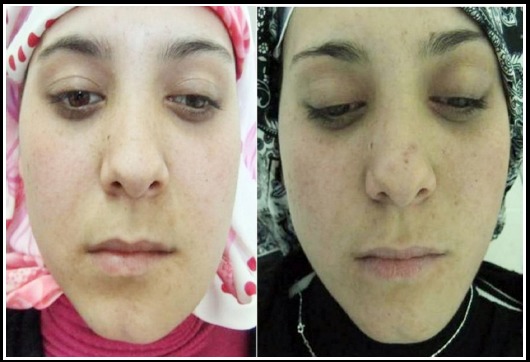
Extraoral photograph; comparison before and after surgery.

No paresthesia was seen during the early and late postoperative period. The patient was followed up for 12 months. The patient was satisfied with both aesthetic and functional results (Fig.4 and 5).

## DISCUSSION

FD is a benign bone lesion characterized by replacement of normal bone by fibro-osseous connective tissue exhibiting varying degrees of osseous metaplasia. Although it is not a common disease, it may cause fracturesin long bones, deformities and severe bone pain.^8^ Differantial diagnosis of FD is considerably based on clinical, radiographic, laboratory and histological findings. The medullary bone is replaced by fibrous tissue, which appears radiolucent on radiographs, with the classically described ground-glass appearance.

Pathological conditions mimicking FD can be classified as other fibro-osseous lesions like; cementoosseous dysplasia, ossifying fibroma, simple bone cysts, cementoma, Paget’s disease, cherubism, hyperparathyroidism, chronic sclerosing osteomyelitis and osteogenic sarkoma. Fibroosseous lesion is a common group of diseases, but the treatment option varies from none to surgical recountering and complete removal. The lesion is most frequently confused with FD is Ossifying Fibroma (OF). Diagnosis of these two lesions should be based on clinical, radiographic and microscopic findings. OF discloses a well-delineated border whereas FD is expansive and diffuse with ill-defined margin.^9^

There is not a definite etiology of FD, but it is believed to arise from abnormal activity in the bone forming mesenchymal tissue.

Approximately 70-80% of FD cases are monostotic. Craniofacial involvement is found in only 10% of the cases in the monostotic variety. Most of the reported FD cases are monostotic type, and maxilla is more frequently affected than mandible. Monostotic type is seen more often in childeren and adults.^4^,^6^ The most common clinical sign of craniofacial FD is swelling while other presenting signs and symptoms depend on the area of involvement. The most commonly involved bones of the craniofacial skeleton are the maxilla and frontal bones.

The clinical and radiographic diagnosis of FD was confirmed with histopathological examinations. Malignant transformation is rare but it may transform into sarcomas. Radiation therapy is important factor for the malign transformation of FD, therefore it is not included in the treatment options for FD.^3^,^6^ Recurrence of FD is rare in adults but more common during the growth period. The risk of recurrence increases in cases where conservative surgery is used and the lesion cannot be totally removed. The surgical treatment of FD ranges from biopsy specimens to modeling osteotomies, bi-maxillary osteotomies and calvarial remodeling. The aim of the surgical therapy for FD is to prevent pathological fractures, control the pain and to reduce bone deformities.^4^,^9^-^11^

Typically FD stabilizes or slows after puberty. Small lesions may only require biopsy to rule out other lesions. Treatment for craniofacial FD is primarily surgery. When the tooth-bearing bones of the jaws (maxillary alveolar bone and mandible) are affected, conservative shaving is the most effective and reasonable treatment. However recurrence and the necessity of additional surgery are not uncommonal when conservative shaving has been used.

In the present case incisional biopsy was carried out twice to eliminate possible malignancy and differentiate the lesion from osteofibrous dysplasia. The affected side of mandible was operated with the treatment protocol through preserving the mental nerve in order to minimize the risk of intraoperative complications including paresthesia and bleeding from neurovascular structures. Paresthesia and bleeding from neurovascular structures was not seen with administration of the respective surgical procedures. More aggressive and extensive surgery including shaving cortex of left mandibular base is avoided during puberty. Additional surgery beyond our initial treatment protocol was not required during the 12 months follow-up period. The patient was satisfied both functionally and estetically. Our treatment protocol through intraoral approach served the patient well. Nevertheless, the combination of extraoral approach including surgical shaving via submandibular dissection and intraoral approach would be performed, if recurrence is observed later on to obtain better esthetic profile.
